# Learning to rank-based gene summary extraction

**DOI:** 10.1186/1471-2105-15-S12-S10

**Published:** 2014-11-06

**Authors:** Yue Shang, Huihui Hao, Jiajin Wu, Hongfei Lin

**Affiliations:** 1 School of Computer Science and Technology, Dalian University of Technology, Dalian, China; 2 College of Computing & Informatics, Drexel University, Philadelphia, PA, USA; 3 School of Information Technology, York University, Toronto, Canada

## Abstract

**Background:**

In recent years, the biomedical literature has been growing rapidly. These articles provide a large amount of information about proteins, genes and their interactions. Reading such a huge amount of literature is a tedious task for researchers to gain knowledge about a gene. As a result, it is significant for biomedical researchers to have a quick understanding of the query concept by integrating its relevant resources.

**Methods:**

In the task of gene summary generation, we regard automatic summary as a ranking problem and apply the method of learning to rank to automatically solve this problem. This paper uses three features as a basis for sentence selection: gene ontology relevance, topic relevance and TextRank. From there, we obtain the feature weight vector using the learning to rank algorithm and predict the scores of candidate summary sentences and obtain top sentences to generate the summary.

**Results:**

ROUGE (a toolkit for summarization of automatic evaluation) was used to evaluate the summarization result and the experimental results showed that our method outperforms the baseline techniques.

**Conclusions:**

According to the experimental result, the combination of three features can improve the performance of summary. The application of learning to rank can facilitate the further expansion of features for measuring the significance of sentences.

## Background

Genome studies have received a tremendous boost in recent years. Thousands of literature articles have been published to present the discovery of genes from different species and their functions, characteristics, expression, and so forth. However, for other researchers, obtaining knowledge for a specific gene requires tremendous research and is quite time and energy consuming.

Nowadays, there are many biomedical researchers engaged in the establishment and maintenance of biomedical databases. For example, Entrez Gene is a gene database developed and maintained by the National Center for Biotechnology Information (NCBI). This database contains information on all aspects of genes, such as full name, resource, type, description, and so forth. According to the statistics in September 2010, there were almost 7 million records in Entrez Gene, distributed among 7,300 taxa [1]. Part of the data contains a field of gene summary information that can facilitate the researchers in obtaining knowledge about the gene. However, most genes do not have the summary tag for description, which is marked by researchers manually. It is arduous to mark a biological gene database with such a vast amount of data. If gene summaries can be generated, this will be convenient for researchers. In order to help understand specific gene information, researchers began to focus on the research and development of a gene information retrieval system or gene automatic summarization system.

Document summarization has been studied for years to extract important information from the documents and to rank the sentences in proper order to save the readers' time and energy.

Most of the existing literature in multidocument summarization techniques focuses on sentence selection using similarity between sentence and query. Various text, syntactic and semantic features have been used for this task, including term frequency, position in which the sentence appears in the document and paragraph, cue words, title, and so forth. Specifically, Luhn tries to solve the problem using term features [2]. Edmundson and colleagues combine four features - term frequency, co-occurrence with title, position and cue words - to generate summaries. Erkan and Radev [3] and Mihalcea and Tarau [4] deem sentence selection a classification problem. Plaza and colleagues proposed a graph-based multidocument summarization method, using UMLS to identify concepts and semantic relations, and then to construct a rich semantic representation of the documents [5]. He and colleagues take a different perspective from data reconstruction and propose a novel framework called Document Summarization based on Data Reconstruction [6]. Specifically, their approach generates a summary that consists of those sentences which can best reconstruct the original document.

Ling and colleagues exploit a gene summary system that is based on biomedical structured data and uses machine learning methods to extract six attributes of a gene, namely gene products, DNA sequence, and so forth [7,8]. In Ling's work, sentences are sorted considering the relevance to the gene's property and the position in which they appear. Jin and colleagues take advantage of the chi-square distribution to find subject terms differing from the general biomedical texts in the description of a gene, and characterize the importance of sentences including these subject terms, using the gene ontology feature and PageRank graph feature [9].

In this paper, we propose a summarization method based on learning to rank. In our method, three kinds of features are developed to describe the sentence weight for sentence ranking. We use learning to rank to obtain the feature weight and the top ranked sentences are collected to generate the gene summary. In our experiment, we use MEDLINE corpus for the experiment and gene description in the Entrez Gene database as a reference. The next section will give a brief introduction about related works, and the following section will describe the features we use and our learning to rank model. We then discuss our experiment settings and result analysis. Finally, we present concluding remarks.

## Methods

In this section we report on the construction of the summarization system, and we describe each step in more detail in the following sections.

This paper presents a method for generating gene summaries from biomedical scientific literature. The method takes three important features into consideration, and we use the learning to rank algorithm to observe the contribution of each feature.

**Figure 1:**
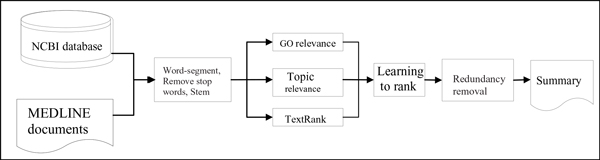
**Framework for gene automatic summarization**. NCBI, National Center for Biotechnology Information.

First we collect the target genes from the NCBI database and collect the relevant document for each target gene from Medline. Our algorithm is based on the following steps to generate gene summaries: preprocess documents in a training set and a test set, such as sentence border identification, word segmentation, stemmer and removing stop words; calculate each sentence's gene ontology relevant score, topic relevant score and TextRank score; for sentences in the training set, compare the similarity between them and reference summaries, and then give a score from 0 to 4 using the recall-oriented understudy for gisting-evaluation (ROUGE) toolkit; for the training set, apply the learning to rank algorithm and gradient descent to learn the weight of features; and, after several iterations, obtain the weight vector. Next we rank sentences in the test set. Finally, through redundancy removal, we can generate the gene's summary. The whole system is illustrated in Figure 1.

## Feature selection

### Gene ontology relevance

The Gene Ontology project (GO) is a biomedical database used to normalize all species' genes and the properties of gene products. The ontology covers three domains: cellular component, molecular function and biological process. The cellular component describes a cell's composition and its extracellular environment. Molecular function represents elemental activities of a gene product at the molecular level, such as binding or catalysis, Biological process describes operations or sets of molecular events with a defined beginning and end, pertinent to the functioning of integrated living units: cells, tissues, organs and organisms.

Every ontology term in the GO includes the following parts: gene ontology name, unique tag composed of letters and figures, and references to resources. The GO is structured as a directed acyclic graph, and each term has defined relationships to one or more terms. The relationships comprise 'is a' and 'part of' relations.

The GO annotation database labels gene products with the GO term. Every GO product is annotated with the GO name, annotated basis, annotated organization and annotated time. Table 1 presents an example of the GO annotation information for the gene actin.

**Table 1:** Gene ontology annotation data

Gene product	Actin, alpha cardiac muscle 1, UniProtKB:P68032
GO term	Heart contraction
GO	0060047 (biological process)
Evidence code	Inferred from Mutant Phenotype (IMP)
Reference	PMID 17611253
Assigned by	UniProtKB, June 6, 2008

GO, Gene Ontology project.

Each gene is unique because of its structural component and function, so the generated summary should include the gene-specific features. GO terms exactly reflect the gene's own property that distinguishes it from others. This is the property our paper makes use of to search for these terms' occurrence in candidate sentences and compute the sentences' GO relevance score. Namely, we prefer sentences that include specific GO terms about the gene. A gene can be annotated by one or several GO terms. Table 2 presents a segment of the GO annotation database that is also a part of the corpus we used in our experiment. From this table we can conclude that the gene AT2G01050 (GeneID: 814629) has several GO terms.

**Table 2:** Gene ontology annotation corpus for AT2G01050

tax_id	GeneID	GO_ID	GO_term	Category
3702	814629	GO:0005575 ND	cellular_component	Component
3702	814629	GO:0003676 IEA	nucleic acid binding	Function
3702	814629	GO:0008150 ND	biological_process	Process
3702	814629	GO:0008270 IEA	zinc ion binding	Function

GO, Gene Ontology project.

The GO relevance score is computed according to GO annotation information. First, given a target gene, we look for corresponding GO terms according to the gene2go data provided by Entrez Gene. Next, we pre-process candidate sentences of the target gene by word segmentation, stemming and stop-word removal. Additionally, frequencies of GO terms are computed and we score the sentences according to the GO score. For a gene, the algorithm procedure for computing the GO relevance score is as follows:

(a) Stem and remove stop words for the GO annotation information;

(b) Segment, stem and remove stop words for the gene's candidate sentences;

(c) For each candidate sentence $S_{k}$, *k *= 1, 2, ..., *n*. Firstly, set the GO relevance score to 0 - that is, $GOScore\left(S_{k}\right)=0$. For each word *w *in sentence $S_{k}$, if the GO term set includes this word, then $GOScore\left(S_{k}\right)+=1$;

(d) Finally,$GOScore\left(S_{k}\right)=GOScore\left(S_{k}\right)/length\left(S_{k}\right)$, where $length\left(S_{k}\right)$ represents the count of words in the sentence $S_{k}$ after pre-processing and removing stop words.

### Topic relevance

In 2003 Blei and colleagues proposed the Latent Dirichlet Allocation (LDA), which is a generative model and can represent the document set and other discrete datasets as topics [10]. At present, the LDA model has been used in many text-relevant fields, such as text classification and information retrieval [11-13].

LDA is an unsupervised learning algorithm and can recognize the latent topics of the document set, without using training data [14]. By using LDA, documents can be represented by the distribution of topics, while topics can be represented by the distribution of words. By applying a topic model to the data, the most related topic is selected from all of the topics about the gene and the words under that topic are used as features for topic relevance.

LDA is a generative probabilistic model of a corpus. The basic idea of LDA is that documents are represented as a distribution of latent topics, where each topic is characterized by a distribution over words. LDA is a directed probability graph model, including three layer structures: word, document and document. LDA can model the topic information in document sets. For a given document set, the LDA model represents each document as a set of topics and each topic is represented by word multinomial distributions.

Recently, the LDA model has been studied in the field of natural language processing and intelligent information processing. Meanwhile, researchers are also performing topic detection using the LDA model in the document summarization field, but it has not been applied to the biomedical field.

In this paper, we will make use of LDA to find the implicit topics in a gene's relevant documents. For all genes' descriptions, we regard these documents as sets of topics. Using the LDA model, we are aiming to mine the gene's topics and obtain the topic words, as Table 3 shows. Finally, we compute the candidate sentences' relevant degree with these topic words. The algorithm used is as follows:

**Table 3:** Topic terms for gene summary

protein	family	encode	member
gene	function	membrane	acid
provide	involve	chromosome	cell
complex	variant	receptor	kinase
conserve	belong	isoform	role
human	transcript	subunit	domain

(1) Segment, stem and remove stop words for the gene's candidate sentences;

(2) For each candidate sentence $S_{k}$, *k *= 1, 2, ..., *n*. At beginning, let $LDAScore\left(S_{k}\right)=0$. For each word *w *in the sentence $S_{k}$, if the topic word set concludes this word, then $LDAScore\left(S_{k}\right)+=1$;

(3) Finally, $LDAScore\left(S_{k}\right)=LDAScore\left(S_{k}\right)/length\left(S_{k}\right)$, $length\left(S_{k}\right)$ represents the sum of words in sentence $S_{k}$ after pre-processing and removing stop words.

### TextRank

TextRank is a graph-based method that computes the importance of sentences [4]. Sentences are regarded as nodes in the graph, while similarities among the sentences are regarded as edges between these nodes. TextRank is similar to PageRank. When node B is connected with node A, this means that node B has voted for node A. Meanwhile, the vote is represented by the similarity between the nodes. The more similar node B is to node A, the more important the vote is from node B to node A. Additionally a node with a higher score will give a more authoritative vote. When a node gets many votes, this means the node is very important and will have a higher score. Conversely, PageRank just analyzes hyperlinks between web pages - a page is either connected with another page or not; but in our method the edge represents the similarity between nodes. PageRank is improved by TextRank by adding a weight to the edge, namely the TextRank weighted graph model. In the TextRank model, the importance of a node is related to the number of votes it obtains, the importance of nodes voting it and similarity between them.

According to the above theory, we can describe the text as a weighted graph *G *= (*V, E*), where *V *is the set of nodes and *E *is the set of edges with V*V. The importance of node *V_i _*is then defined by Equation (1):

(1) $$S\left(V_{i}\right)=\left(1-d\right)+d\times \underset{j\in In\left(V_{i}\right)}{\sum }\frac{w_{ji}}{\left|Out\left(V_{j}\right)\right|}S\left(V_{j}\right)$$

Here we do not consider the direction of the edges. The out-degree of a node in the graph is equal to its in-degree - that is, Out (*V_j_*) = In (*V_j_*) - and *d *is a parameter that can be set between 0 and 1, which has the role of integrating into the model the probability of jumping from a given vertex to another random vertex in the graph.

To apply TextRank in our work we first need to build a graph associated with the document, where the graph vertices are representative or the units to be computed. For the goal of computing the sentence's TextRank score, a vertex is added to the graph for each sentence in the documents. The first step is thus to identify the sentence unit in the documents.

The procedure of the algorithm is as follows:

(1) Detect sentences in the document set, each sentence corresponds to a node in the graph;

(2) Compute similarities between every two sentences, we employ cosine similarity as Equation (2) shows:

(2) $$E_{i,j}=Cos\left(S_{i},S_{j}\right)=\frac{\overset{t}{\underset{k=1}{\sum }}w_{i,k}\times w_{j,k}}{\sqrt{\overset{t}{\underset{k=1}{\sum }}w_{i,k}^{2}}\times \overset{t}{\underset{k=1}{\sum }}w_{j,k}^{2}}$$

w=tf(w)×idf(w), *tf*(*w*) is the frequency of word *w *in the sentence and $idf\left(w\right)=1+\text{log}\left(\frac{N}{n_{w}}\right)$, where *N *is the total number of documents and $n_{w}$ is the sum of documents containing word *w*.

(3) Initialize each node's value in the graph to an arbitrary value between 0 and 1;

(4) Iterate Equation (1) until all of the nodes' different values are smaller than a given threshold. These values are their TextRank scores.

### Learning to rank

There are two types of automatic summarization techniques: extractive summary and abstractive summary. There are many technical difficulties for an abstract summary to realize, so we adopted an extractive summary technique to generate a gene's summary. Given a gene, the system will grade those relevant sentences and find the most representative sentences. The process can be regarded as a sentence ranking problem.

With the development of information retrieval techniques, more features are brought into the ranking algorithm. Learning to rank combines information retrieval techniques and machine learning theory, and its goal is to obtain a ranking model from the training set using various algorithms and ranking documents in the test set [15].

When applied to automatic summarization, the task of learning to rank is as follows: for a given query and its relevant documents, the ranking function would give a score to every document. In the training set, each of the relevant documents has a definite score. The score represents the relevance degree of the document to the query, and can be explicitly or implicitly given by humans. Through minimizing the loss function and a series of iterations, a ranking function is created in the training set, such that the model can precisely predict the ranking lists in the training data. We can then use it for the test set.

Recently, the learning to rank algorithm has been drawing broad attention in the machine learning community. Several methods have been developed and successfully applied to document retrieval, such as pointwise, pairwise, listwise, and so on. In this paper, we employ what we call the listwise approach [15], in which document lists are used as instances in learning, and the listwise loss function is called ListNet, with the neural network as the model and gradient descent as the algorithm. Next, we will provide a detailed description about using the listwise method to rank candidate sentences.

First, given a query set $Q=\left(q_{1},q_{2},\ldots ,q_{n}\right)$. Each query *q_i _*corresponds to a gene and has a candidate sentence list, $s_{i}=\left(s_{1},s_{2},\ldots ,s_{n}\right)$, of which $s_{j}$represents the *j*th candidate sentence of *q_i_. n_i _*is the sum of its candidate sentences *s*.

For each candidate sentence list, $s_{i}=\left(s_{1},s_{2},\ldots ,s_{n}\right)$, there is a corresponding list, $y_{i}=\left(y_{1},y_{2},\ldots ,y_{n}\right)$, describing the candidate sentence's importance, with $y_{i,j}$ the score of sentence $s_{i,j}$. The importance is defined according to cosine similarity between the sentence and description *d_i _*of gene *q_i_*:

(3) $$y_{i,j}=cos\left(s_{i,j},d_{i}\right)=\frac{\overset{t}{\underset{k=1}{\sum }}s_{ij,k}\times d_{i,k}}{\sqrt{\overset{t}{\underset{k=1}{\sum }}s_{ij,k}^{2}}\times \overset{t}{\underset{k=1}{\sum }}d_{i,k}^{2}}$$

The assumption of Equation (3) is that the more similarity between candidate sentence $s_{i,j}$ and $q_{i}$'s description *d_i_*, the more appropriate is $s_{i,j}$ as the summary sentence.

In the process of the training model, the training set can be denoted as $\Gamma ={\left\{x_{i},y_{i}\right\}}_{i=1}^{n}$. There are *n *queries for each query *q_i_*, and there is a feature vector list, $x_{i}=\left(x_{1},x_{2},\ldots ,x_{n}\right)$. A feature vector $x_{i,j}$ is created from each query-sentence pair (*q_i_, s_i,j_*); *i *= 1, 2, ..., *n*; *j *= 1, 2, ..., *n_i_*. Each list of features $x_{i}=\left(x_{1},x_{2},\ldots ,x_{n}\right)$ and the corresponding list of scores $y_{i}=\left(y_{1},y_{2},\ldots ,y_{n}\right)$ then form an 'instance'.

Given the feature vector list $x_{i}$, ranking function *f *will calculate a relevance score *f*($x_{i}$). Then for each feature vector list, we can get a relevance score list $z_{i}=\left(z_{1},z_{2},\ldots ,z_{i}\right)$ = (*f*($x_{i}$,1), *f*($x_{i}$,2), ..., *f*($x_{i}$,$n_{i}$)). The goal of learning to rank is to minimizing the sum of the training set's loss function   □

$$\overset{n}{\underset{i=1}{\sum }}L\left(y_{i},z_{i}\right)$$

where *L *is the loss function to be optimized.

To obtain the objective function, this paper employs a gradient descent algorithm to optimize loss function. We call this method ListNet. The gradient descent algorithm is an important learning paradigm, which is a strategy for searching enormous hypothesis space and can satisfy the condition of continuous parameter assumption and error differentiation for arguments.

We then use the ranking function to assign the score for the sentences in the test set. The input of the ranking function is the three features in Section A. For example, given a gene A and a set of sentences containing A, we can calculate a score for each sentence. Then we rank the sentences and obtain the top *k *for the next step. We call the learning problem described above the listwise approach to learning to rank.

The specific idea of the algorithm is shown in Figure 1. In our experiment, we obtain the feature weight vector (0.1, 0.2, 0.7) that has the best performance after 1,000 iterations.

#### Redundancy removal

A good summary should not only contain as much diverse information as possible for a gene, but also with as little redundancy as possible. For many well-studied genes, there are thousands of relevant papers and most information is redundant. Hence it is necessary to remove redundant sentences before producing a final summary. Here the idea of redundancy removal is that when a sentence is similar to a sentence selected in the summary, then the sentence should be punished. Hypothesize that *S *is the last sentence set of the generated summary and *C *is the candidate sentence set; the algorithm of redundancy removal is then as follows:

**(1) **In the initial state, *S *=  Φ, *C *= (*s_i _*|*I *= 1, 2, ..., *n*) concludes *n *candidate sentences. Score these sentences using the function and feature weight vector that we have obtained in the former step;

**(2) **Rank sentences according to their score;

**(3) **Select sentence *s_i _*with the highest score_, _move it from *C *to *S*. Meanwhile, update scores of sentences in *C *with Equation (4):

(4) $$Score\left(s_{j\;}\right)=Score\left(s_{j}\right)-\omega \times sim\left(s_{i},s_{j}\right)$$

where ω > 0 is the punishment parameter, here set as an empirical value 1.0. *sim*(*s_i_, s_j_*) is the cosine similarity between *s_i _*and *s_j_*.

**(4) **Repeat steps (2) and (3) until the length of sentences in *S *reaches the length set before.

## Results

### Data collection

Entrez Gene is a gene database developed and maintained by the NCBI. The database reports multiple types of information about genes, including nomenclature, summary descriptions, accessions of gene-specific and gene product-specific sequences, chromosomal localization, reports of pathways and protein interactions, associated markers and phenotypes. In this paper, we use the summary description in the property 'Entrezgene-summary' as the gene's reference summary to be compared with the summary we generate. Meanwhile, we obtain all related Medline PubMed IDs from gene2pubmed data provided by Entrez Gene. By using these IDs, we can extract each gene's related documents in the Medline database as the candidate document set to generate the gene's summary.

In the Entrez Gene database, there are 46,362 genes related to human. Among those genes, we selected 3,000 genes having the description summary to experiment with. These genes are deemed the ground truth, and we can compare the summaries we generated with these description summaries. Ranking GeneIDs, we exploit the first 2,000 genes as a training set and the following 1,000 genes as a test set. Although the length of reference summaries varies, most of them contain five sentences. To produce a summary of similar length, we decided to select five sentences in our system.

### Evaluation metrics

A large-scale evaluation is performed using ROUGE metrics. ROUGE is an evaluation package commonly used to automatically evaluate both single-document summarization and multidocument summarization systems [16]. ROUGE measures the quality of summary we generate by counting its overlapping units, such as the *n*-gram, word sequences and word pairs, with reference summary. Among all of the evaluation metrics in ROUGE, ROUGE-N and ROUGE-SU generally perform well in evaluating multidocument summarization according to Lin and Hovy [16]. We evaluate our summary with the metrics ROUGE-1, ROUGE-2 and ROUGE-SU4. ROUGE-N models *n*-gram-based co-occurrence statistics, where N stands for the length of the *n*-gram. ROUGE-SU4 models skip-bigram plus unigram-based co-occurrence statistics; that is, pairs of words allowing for no more than four words.

It is important to note that ROUGE does not consider the semantic similarity. Since it only counts the lexical matching, when there are two summaries that have similar meaning but use different words, ROUGE may give two different evaluation results.

### Compared methods

To evaluate the summarization performance, different types of summaries have been generated, we have selected two baselines: random sentences, selecting five sentences randomly from the gene's candidate sentences; and MEAD

[17], the most elaborate publicly available platform for multilingual summarization and evaluation. MEAD's source and documentation can be downloaded [18]. The MEAD platform implements multiple summarization algorithms such as position-based, centroid-based, largest common subsequence and keywords. MEAD has been used in numerous applications, ranging from summarization for mobile devices, to webpage summarization within a search engine, to novelty detection. The latest edition of MEAD is version 3.12. Here we use the default setting that extracts sentences according to three features: centroid, position and length. The length of summary is set to 5.

### Experimental results

ROUGE can generate three kinds of scores: the F-measure, precision and recall. In this experiment, our method is always taking the lead among the three types of score. We only use recall to compare different approaches. As stated in Section B, the recall of three ROUGE metrics is shown in our experimental results: ROUGE-1, ROUGE-2 and ROUGE-SU4.

Table 4 presents the ROUGE evaluation results. Our learning to rank method is presented with respect to three features. As shown by the highest ROUGE scores (Table 4 bold type), learning to rank obviously reports higher ROUGE scores than the other summarizers.

**Table 4:** Performance comparison between different systems

Method	ROUGE-1	ROUGE-2	ROUGE-SU4
MEAD	0.39	0.08	0.14
Random	0.31	0.05	0.11
LTR	**0.46**	**0.1**	**0.17**

Bold data represent the highest recall-oriented understudy for gisting-evaluation (ROUGE) scores. LTR, learning to rank with respect to three features.

To test the impact of the features' combination with respect to learning to rank, we also performed three groups of experiments. Because of space constraints, however, only one group of results is explored here, as Table 5 presents. In this group, we first conducted TextRank. Then, on the basis of these results, we add the other two features respectively. For legibility reasons, only the ROUGE-1 score is shown. It may be observed from Table 5 that the last combination behaves the best when all three features are used in learning to rank. In contrast, the latter combination (that is, TextRank and GO) achieves slightly better results than the former combination (that is, TextRank and LDA).

**Table 5:** Contribution of features of TextRank, LDA and GO to the experimental results

Method	ROUGE-1
TextRank	0.36
TextRank + LDA	0.4
TextRank + GO	0.42
TextRank + GO + LDA	0.46

GO, Gene Ontology project; LDA, Latent Dirichlet Allocation; ROUGE, recall-oriented understudy for gisting-evaluation.

## Discussion

In this section, we will discuss the results. First, we discuss the results of the final evaluation and compare our method with the other two summarizers. It can be concluded from Table 5 that our method outperformed the two baseline techniques. One reason for this is that we applied three effective features. The gene ontology relevance score prefers the sentences with specific ontology information about a gene; topic relevance rewards sentences including gene topics; and TextRank would assign higher scores to representative sentences. Thus, if a sentence contains the gene ontology we desire, contains phrases about gene topics and also has a higher similarity with other related sentences, it will more probably be chosen as a summary sentence. The use of three features along with learning to rank allows the system to identify more specialized and representative sentences as summaries. MEAD does not use any biomedical features; instead, it only selects sentences that are centers of the cluster of documents. MEAD therefore cannot reflect the importance of a sentence about a target gene.

Meanwhile, we also analyzed the reference summary. Unsurprisingly, the sentences in the reference model often included the gene's GO terms and its representative descriptions, such as function, species, variance, and so on, which is similar to the topics that we obtained using the LDA model. Because ROUGE metrics are based on the number of word overlaps, this model frequently awards summarizers containing the same terms as reference summaries.

Furthermore, we can conclude that larger promotions are gained in ROUGE-2 and ROUGE-SU4 than in ROUGE-1. This is because many background words (for example, gene, protein, cell) also appeared frequently as unigrams in the reference summaries.

We also analyzed the results of the features' combination, aiming to analyze the contribution of each feature to the task. We can observe the three features' impact on the experiment's results from Table 5. These results show that the combination can improve the summary's performance via bringing in three biomedical features along with learning to rank. At the same time, the GO feature has a better effect on the summaries than the LDA topic model. After observing the reference summaries, we found that different genes cover different topics and the summaries we generated cannot catch these topics accurately. But in the light of gene terms, there are definite words for each gene, so the GO feature has the better result. In future work, we can carry out some experiments to check the amount of impact of different LDA topics on the summaries.

After reviewing the results, we believe that the generated summaries can amalgamate the important information of a gene from multiple documents and the proposed method has a promising performance compared with the baseline techniques. This model can help biomedical researchers to have a quick understanding of a gene and decrease the workload of annotators.

## Conclusions

In this paper, we propose a multidocument summarization focused on the gene summary domain. We conducted three different features - gene ontology score, topic relevant score and TextRank score - to describe the characteristics of a gene. Learning to rank is applied to model the contribution of each feature from the training dataset. We conducted the experiment on the Entrez Gene database developed by the NCBI and the Medline database. The experimental results showed that the summaries generated by our method have a better performance than those from the baseline methods. At the same time, learning to rank contributes to useful feature expansion for ranking candidate sentences, and will facilitate the import of features evaluating the importance of sentences.

In the future, we can make an in-depth study of introducing more efficient features into ranking sentences, such as BM25, a linguistic model to find effective features or their combination. Moreover, we will add the query-driven idea to our system, in order to fulfill the user's information need.

## Abbreviations

GO, Gene Ontology project; LDA, Latent Dirichlet Allocation; NCBI, National Center for Biotechnology Information; ROUGE, recall-oriented understudy for gisting-evaluation.

## Competing interests

The authors declare that they have no competing interests.

## Authors' contributions

YS carried out the overall algorithm design and experiments. HH participated in the draft writing. JW contributed to algorithm design and implementation. HL contributed to the algorithm design. All authors read and approved the final manuscript.

## Declarations

Publication of this article has been funded by the Natural Science Foundation of China (No. 60673039, 60973068, 61277370, 61070098, 61272373), the National High Tech Research and Development Plan of China (No. 2006AA01Z151), Natural Science Foundation of Liaoning Province, China (No. 201202031), State Education Ministry and The Research Fund for the Doctoral Program of Higher Education (No. 20090041110002).

This article has been published as part of *BMC Bioinformatics *Volume 15 Supplement 12, 2014: Selected articles from the IEEE International Conference on Bioinformatics and Biomedicine (BIBM 2013): Bioinformatics. The full contents of the supplement are available online at http://www.biomedcentral.com/bmcbioinformatics/supplements/15/S12.
